# Prevalence of Keratoconus in a Refractive Surgery Population

**DOI:** 10.1155/2018/5983530

**Published:** 2018-09-06

**Authors:** Abdulrahman Mohammed Al-Amri

**Affiliations:** College of Medicine, King Khalid University, Abha, Saudi Arabia

## Abstract

**Objective:**

This study examined the prevalence of keratoconus among patients who were interested in undergoing refractive surgery. Corneal tomography measurements were used to help detect keratoconus.

**Methods:**

Adult subjects who presented to the private hospital Cataract and Refractive Surgery Unit (Abha, Saudi Arabia) for refractive surgery evaluation were considered for inclusion in this cross-sectional, retrospective study. All subjects were from the Aseer province, a southern, high-altitude region in Saudi Arabia, and presented between January and December 2017. The incidence of keratoconus and other refractive surgery contraindications were examined.

**Results:**

A total of 2931 patients were considered for inclusion in analyses. Of these, 2280 patients (77.8%) were not candidates for refractive surgery. These 2280 patients had a mean age of 24.1 ± 6.6 years and 1231 patients (54.0%) were male. Of the subjects who did not undergo refractive surgery, 548 (24%) had keratoconus, 400 (17.5%) were keratoconus suspects, 344 (15.1%) had thin corneas, 321 (14.1%) had high myopia, and 52 (2.3%) had a high astigmatism. An additional 479 subjects (21%) were candidates for refractive surgery, but chose not to undergo a procedure.

**Conclusion:**

The incidence of keratoconus in Saudi Arabian refractive surgery prospects was 18.7%. Keratoconus was the most common reason for not performing refractive surgery and accounted for 24.0% of cases in which surgery was not performed.

## 1. Introduction

Keratoconus is a cone-shaped protrusion of the cornea that was named using the Greek terms “kerato” and “konos,” which mean “cornea” and “cone,” respectively. Keratoconus begins as a corneal thinning and results in a corneal bulge. As a result, light is irregularly refracted through the cornea (astigmatism), which is apparent during retinoscopy. As the cornea progressively steepens, astigmatism becomes more severe and visual acuity subsequently decreases [1, 2]. Keratoconus can be treated in several ways, depending upon disease stage. Contact lenses can be used to correct vision in early disease stages, but this solution often becomes inadequate and some patients cannot wear contact lenses. Therefore, some treating physicians choose to implant intrastromal corneal ring segments to flatten and stabilize the cornea, to improve vision, and, in some cases, enable the use of contact lenses [3]. However, in advanced cases, corneal transplantation (full or partial thickness) is often needed. In recent years, collagen cross-linking procedures have been used to successfully stabilize and reshape the cornea, resulting in long-term vision improvements [4–7]. Additionally, successfully treated patients may avoid corneal transplantation.

Keratoconus is of particular significance in refractive surgery candidates because operating on an eye with undetected keratoconus is a major cause of postoperative corneal ectasia [8–11]. The underlying cause of this progressive, bilateral, ectatic condition remains unknown. However, genetics are believed to play a role because up to 20% of patients with keratoconus have a positive family history for condition [12, 13] and a family history of keratoconus is a risk factor for developing the condition [14–18]. Allergy-induced mechanisms may also play a role because the risks of developing keratoconus are higher [19–21] and age of onset is lower [22] in patients with allergic or atopic disease.

Many studies have been performed around the globe to assess the incidence of keratoconus. The overall incidence of keratoconus is estimated at 50 cases per 100,000 people (0.05%) [23]. However, this widely varies by geographical region, as summarized in Table 1. For example, a study on a Russian population reported an incidence of 0.2 cases per 100,000 people (0.0002%) [24], while a study on a central Indian population reported an incidence of 2300 cases per 100,000 people (2.3%) [25]. The incidence of keratoconus in the United States has been estimated to be 54.5 cases per 100,000 people (0.06%) [17]. However, keratoconus detection rates can vary based on investigative method used and sample size examined [26, 27]. Corneal topography is the gold standard for detecting keratoconus. Therefore, prior studies that investigated keratoconus incidence in refractive surgery prospects, all of whom undergo corneal topography studies, are of particular importance. These studies found an incidence of 3.0% and 5.5% for keratoconus and suspected keratoconus in a Caucasian population, respectively [10]. In another study on a Yemenite population, the incidences for keratoconus and suspected keratoconus were 18% and 10%, respectively [28]. The current study also used a topography-based approach to examine the incidence of keratoconus in patients presenting to our clinic seeking refractive surgery. It should be noted that all included subjects were from a high altitude region of Saudi Arabia.

## 2. Materials and Methods

This study was reviewed and approved by the private hospital human research Ethics Committee (EC). Written informed consent was obtained from all patients. All study conduct adhered to the tenets of the Declaration of Helsinki.

### 2.1. Study Subjects

This cross-sectional study examined data that were retrospectively obtained from patient files. All subjects presented to the private hospital Cornea and Refractive Surgery Unit (Abha, Saudi Arabia) between January and December 2017 seeking refractive surgery. All subjects had undergone standard ophthalmologic examination and corneal tomographic assessment with the Pentacam HR system (Oculus, GmbH, Wetzlar, Germany). Additionally, contact lens users had not worn their lenses for at least 3 weeks prior to examination. Patients were excluded from the study if they were younger than 18 years of age or if they had a history of ocular surgery or trauma. Patients were also excluded if they had incomplete medical records.

### 2.2. Data Collection

Patient demographic, corneal topographic, and medical data were collected from standard examinations performed to determine refractive surgery eligibility. Reasons for not undergoing refractive surgery were identified, with specific focus on the presence/absence of keratoconus. Subjects were classified as having keratoconus if at least two of the following criteria were met: corneal thickness <500 *µ*m, asymmetric bowtie on corneal topography map, corneal steepening ≥47 D, skewed radial axis >21°, posterior elevation >20 *µ*m, and inferior-superior (I-S) asymmetry >1.4 D. Subjects were classified as keratoconus suspects if one of the following criteria was met: corneal thickness <450 *µ*m, asymmetric bowtie on corneal topography map, corneal steepening ≥48 D, posterior elevation >25 *µ*m, or I-S asymmetry >1.6 D.

### 2.3. Data Analyses

Continuous data are presented as mean ± standard deviation. Frequency and prevalence data are presented as *n* (%). Data normality was verified using a standard normality test, continuous data were compared using unpaired Student's *t*-tests and categorical data were compared using chi-square tests. Correlations between subject characteristics and keratoconus frequency were examined using Pearson's correlation analyses. All statistical analyses were performed using the Statistical Package for Social Sciences (SPSS) software (ver. 20, SPSS, Inc., Chicago, IL). Statistical significance was defined as *p* < 0.05.

## 3. Results

A total of 2931 patients were included in this study. Of these, 2280 patients (77.8%) were not candidates for refractive surgery. These subjects had a mean age of 24.1 ± 6.6 years (range: 18–52 years) and 1231 (54.0%) were female. As summarized in Table 2, the most common reasons for not undergoing surgery were keratoconus (548 patients [24.0%]), keratoconus suspect (400 patients [17.5%]), thin corneas (344 patients [15.1%]), and high myopia (321 patients [14.1%]). An additional 479 patients (21.0%) were refractive surgery candidates, but chose not to undergo a procedure.

The overall prevalence of keratoconus in our study sample was 18.7% in all patients and 24.0% in patients who did not undergo refractive surgery. Slightly more than half of the patients with keratoconus were female (284 of 548 patients [52.0%]) and 22.4% of patients had a family history that was positive for keratoconus. The gender distribution was not significantly different between patients with and without keratoconus (1196 of 2384 patients [50.1%]). However, the proportion of female patients was significantly higher in patients with keratoconus (52.0%) than in those who did not have keratoconus (765 of 1733 patients [44.1%], *p*=0.001). Patients with keratoconus were distributed across age groups as follows: 142 patients (26.0%) between 18 and 20 years of age, 182 (33.3%) patients between 21 and 31 years of age, 168 (30.7%) patients between 32 and 42 years of age, and 65 patients (11.9%) between 43 and 53 years of age. This age distribution was not significantly different than in patients without keratoconus (*p*=0.001). However, there was a moderate negative correlation between keratoconus presence and patient age (*r* = −0.612, *p*=0.01).

Similarities and differences between normal, keratoconus suspects, and keratoconus subjects were examined (Table 3). Keratoconus was not found in 1130 subjects (651 procedures plus 479 patients who were refractive surgery candidates and did not undergo a procedure). A total of 400 subjects (17.5%) were keratoconus suspects and 548 subjects (24.0%) had keratoconus. Keratoconus subjects (29.3 ± 5.1 years) were significantly older than both keratoconus suspects (24.9 ± 3.8 years) and normal subjects (18.6 ± 4.1 years), and keratoconus suspects were significantly older than normal subjects (all *p* < 0.001). Additionally, both the keratoconus suspect (29.3%) and keratoconus suspect (23.2%) groups had a higher percentage of patients with a family history of keratoconus than the normal group (17.4%, *p* < 0.001 and *p*=0.009, resp.). Family history was not significantly different between the keratoconus suspect and keratoconus suspect groups (*p*=0.174). All examined corneal abnormalities were observed more often in subjects with keratoconus than in subjects suspected of having keratoconus (all *p* < 0.001).

## 4. Discussion

The current study assessed keratoconus prevalence among refractive surgery prospects in the Aseer (Asir) province of Saudi Arabia. We found an overall keratoconus incidence of 18.7% (18700 cases/100,000 people), which was higher than the historical range of 0.03–3.18% [13, 16, 17, 22–24, 29–35]. A total of 547 of 2280 patients (24.0%) who did not undergo refractive surgery had keratoconus, making it the most common reason for not undergoing a procedure. Another 399 patients (17.5%) were keratoconus suspects.

Keratoconus is influenced by family history [14–18] and ethnicity [36, 37]. Therefore, we compared our findings to those previously obtained in the Middle East. The keratoconus incidence observed in the current study is higher than that previously reported in the Middle East and other regions. A 2005 Saudi Arabian study [13] found a keratoconus incidence of only 20 cases/100,000 people (0.02%). However, that study relied upon keratometry data to detect keratoconus and only included patients referred to a provincial tertiary ophthalmology department. Because corneal tomography is the gold standard for detecting keratoconus [13, 18, 38], it is possible that this 2005 incidence was artificially low. Our findings (24.0% prevalence) are somewhat in agreement with a recent study that found a 17.5% prevalence of keratoconus among college-age refractive surgery prospects in northern Egypt [39]. It may have been that our methods were more sensitive for detecting keratoconus because our incidence of keratoconus suspects was also higher than that in a college-age Palestinian population (17.5% vs. 8.4%). It is puzzling why presumably healthy, young refractive surgery candidates would have such a high prevalence of keratoconus. Therefore, the findings of the current study and the prior Egyptian study may indicate that keratoconus is more prevalent than believed in some regions. Further study is needed in these populations to confirm and better understand our findings.

Slightly fewer men than women had keratoconus in the current study (48% men). This gender distribution does not agree with prior studies, which found that 55–75% of patients with keratoconus were male [17, 24, 32, 34, 40, 41]. Furthermore, men are at a significantly higher risk for developing keratoconus (odds ratio: 2.3–5.4 [16, 21]) and often develop the condition at a younger age [41, 42] than women. Perhaps our relatively small sample size contributed to our findings regarding gender. However, it should be noted that one Iraqi keratoconus patient study populations was made up of 61.1% women [43].

Patients with keratoconus were evenly distributed across age groups, with the exception of the oldest group (43–53 years of age). This is in agreement with prior studies, which found that most keratoconus patients are diagnosed between 21 and 40 years of age [40]. We also found that patient age was significantly and negatively correlated with keratoconus frequency, which may be related to negative correlation between age and severity that was previously reported [34, 37]. Additionally, 22.4% of our patients with keratoconus had a positive family history for the condition, which is in agreement with prior studies [34, 37].

Our study had several limitations related to its retrospective study design and a relatively small sample size. Further prospective studies with a larger number of patients are needed to confirm and better understand our results. In summary, our population had a very high incidence of keratoconus, which was the cause for not undergoing refractive surgery in 24.0% of patients who were not candidates. Age and gender did not heavily influence keratoconus rates in our study population.

## Figures and Tables

**Table 1:** Epidemiology of keratoconus in various countries around the world.

Study	Country	Prevalence (cases/100,000)	Incidence (cases/100,000)	Age at onset (years)	Keratoconus family history (%)
Assiri et al. [13]	Saudi Arabia	—	20	18.5	16
Hashemi et al. [16]	Iran	4000	—	—	—
Ziaei et al. [29]	Iran	2500	22.3–24.9	—	15
Waked et al. [30]	Lebanon	3330	—	—	12.1
Millodot et al. 2011 [21]	Israel	2340	—	—	22
Shneor et al. [43]^∗^	Israel	3180	—	—	0
Shehadeh et al. [31]	Palestine	1451.6	—	—	—
Jonas et al. [25]	India	2300	—	—	—
Godefrooij et al. [32]	The Netherlands	265	13.3	28.3	—
Nielsen et al. [33]	Denmark	86	1.3	—	—
Pearson et al. [34]^∗∗^	United Kingdom	57, 229	4.5, 19.6	26.5, 22.3	—
Kennedy et al. [17]	United States	54.5	2.0	—	—

^∗^ *n* = 10 subjects with keratoconus. ^∗∗^Data reported for white and Asian populations, respectively.

**Table 2:** Reasons for not performing a procedure in refractive surgery candidates (*n* = 2280 patients).

Reason	*n* (patients)	%
Keratoconus	548	24.0
Chose not to undergo a procedure	479	21.0
Keratoconus suspect	400	17.5
Thin corneas (<450 *µ*m)	344	15.1
High myopia	321	14.1
High astigmatism	52	2.3
Dry eyes	39	1.7
Amblyopia	27	1.2
Unstable refraction	25	1.1
Retinal disorder	22	1.0
Large pupil	13	0.6
History of radial keratotomy	8	0.4
Cataract	2	0.1

**Table 3:** Subject and ocular parameters of normal (LASIK candidate), keratoconus suspects, and keratoconus subjects.

	No KC	KC suspect	KC	*p* (N vs KCS)	*p* (N vs KC)	*p* (KCS vs KC)
*n* (subjects)	1130	400	548			
Subject age (years)	18.6 ± 4.1	24.9 ± 3.8	29.3 ± 5.1	<0.001	<0.001	<0.001
Family history of KC	197 (17.4%)	93 (23.2%)	149 (27.1%)	0.009	<0.001	0.174
Corneal thickness < 500 *µ*m	—	321 (80.25%)	520 (94.8%)	—	—	<0.001
Asymmetric bowtie	—	278 (69.5%)	493 (89.9%)	—	—	<0.001
Corneal steepening ≥ 47 D	—	356 (89%)	531 (96.9%)	—	—	<0.001
Skewed radial axis > 21°	—	160 (40%)	372 (67.9%)	—	—	<0.001
Posterior elevation	—	276 (69%)	487 (88.9%)	—	—	<0.001
I-S asymmetry	—	324 (81%)	504 (91.9%)	—	—	<0.001

N = no KC; KC = keratoconus; KCS = KC suspect; I-S = inferior-superior.

## Data Availability

The data used to support the findings of this study are included within the article.
